# Cross-Sectional and Radiologic Imaging as an Adjunct Teaching Modality in Musculoskeletal Anatomy Education

**DOI:** 10.2106/JBJS.OA.25.00255

**Published:** 2026-06-03

**Authors:** Ameen Suhrawardy, Dylan Moran, Muhammad Waheed, Sayf Al-katib, Rahul Vaidya, Malli Barremkala, Drew D. Moore

**Affiliations:** 1 Department of Orthopaedic Surgery, Wayne State University, Detroit, Michigan; 2 Oakland University William Beaumont School of Medicine, Royal Oak, Michigan; 3 Department of Orthopaedic Surgery, Corewell Health William Beaumont University Hospital, Royal Oak, Michigan

## Abstract

**Background:**

Exposure to musculoskeletal (MSK) imaging is limited in medical education. Radiology integration and module-based learning have been previously shown to improve learning when integrated with anatomy education. This project aims to create, implement, and assess self-directed, image-based modules to enhance visuospatial understanding of MSK anatomy among undergraduate medical students. We hypothesize that students using this resource will show greater anatomical understanding and higher end-of-course examination scores.

**Methods:**

Two modules incorporating interactive interfaces and cross-sectional imaging with MSK anatomy were integrated into the preclinical MSK course at a single U.S. M.D. program. Data on student engagement with the module were compared with performance on the end-of-course written examination and practical examination. Subjective data on the perceived efficacy of the educational resource were collected.

**Results:**

Between the academic years of 2022 and 2025, 114 (23.5%) and 165 (33.8%) second-year medical students completed the optional spine and upper extremity modules, respectively, from 488 total enrolled in the MSK course. Those who completed the spine module and the upper extremity module had a mean 3.5% increase (p < 0.0001) and 3.3% increase (p = 0.00025) in final practical anatomy examination scores compared with those who did not use the modules. Usage of the module did not result in a statistically significant increase in final written examination scores. 98.0% of respondents on the postmodule survey reported that the modules improved their understanding of MSK anatomy.

**Conclusion:**

The results from the collected data show utilization of interactive modules incorporating imaging alongside MSK anatomy specifically improved performance on practical anatomy assessments. Integration of cross-sectional and radiologic imaging in MSK anatomy instruction can improve visuospatial understanding of anatomy among medical trainees and can be an effective method to prepare them for clinical application.

## Introduction

One of the great challenges of orthopaedic teaching is imparting the complex architecture of the musculoskeletal (MSK) system and its interrelationships with neurovasculature and soft tissue. Most medical curricula place emphasis on gross anatomy proficiency for preclinical medical students, including MSK education. Clinically, recognizing pathoanatomy on radiographic and cross-sectional imaging is just as critical as identifying anatomy on a fully dissected human cadaver.

Exposure to MSK anatomy and orthopaedics remains limited in medical curricula, with students reporting lower confidence in MSK imaging and physical examinations^1,2^. This disconnect becomes particularly evident during clinical clerkships, where students are expected to translate their MSK knowledge into interpreting imaging, examining patients, and generating differential diagnoses for MSK complaints^3^. A national-level assessment of clinical confidence found that core preclinical MSK education rarely translated into clinical competency; students disproportionately rated their ability to perform knee, shoulder, and ankle examinations as “below adequate”^2^. Focused educational interventions, such as preclerkship workshops or clinical blocks, significantly improved both confidence and examination performance in MSK regions such as the knee and shoulder (p < 0.01), underscoring that without dedicated, interactive training tied to anatomy education, students often enter clerkships with inadequate preparation for MSK pathology^2^.

Since 2000, most preclinical anatomy curricula have incorporated radiology, but the degree of integration varies widely, with educators determining whether it is taught alongside or directly integrated with gross anatomy courses^4^. Previous studies have shown preclinical radiologic anatomy exposure to enhance students’ understanding of MSK structures in a way that complements traditional gross anatomy teaching^4,5^. Unlike cadaveric dissection, which presents static, idealized anatomy, radiologic imaging introduces students to contextualized anatomic structures and spatial relationships. Studies suggest that early imaging exposure paired with anatomy instruction improves student confidence and performance with MSK pathology during clinical clerkships^6,7^.

The purpose of this project is to create, implement, and assess an educational resource that integrates radiologic imaging alongside MSK anatomy for undergraduate medical students during their MSK instruction. We hypothesize that students who use this resource will demonstrate better understanding of the anatomy and improved performance measured through numerical scores on end-of-course practical examinations.

## Methods

### Study Design

The preclinical MSK course at a single American M.D. program was identified as a medium to design and implement a targeted educational resource. A multidisciplinary team including faculty from the departments of foundational medical sciences (author M.B.), radiology (S.A.), and orthopaedic surgery (D.M.) collaborated to develop 2 educational modules on MSK anatomy and imaging. This study received approval from the university’s institutional review board.

The educational modality used to incorporate these radiologic concepts was an interactive, module-based system integrated into students’ online learning platform. This blended approach promotes active engagement, spatial reasoning, and self-paced learning.

### Module Creation and Administration

Two modules were developed on the H5P module platform and directly integrated into the course page on the institution’s online learning management system, Moodle. The modules are region specific: one for upper extremity anatomy including relevant MSK anatomy of the shoulder, upper arm, elbow, forearm, wrist, and hand and one for spine, back, vertebral column, and spinal cord.

**Fig. 1:**
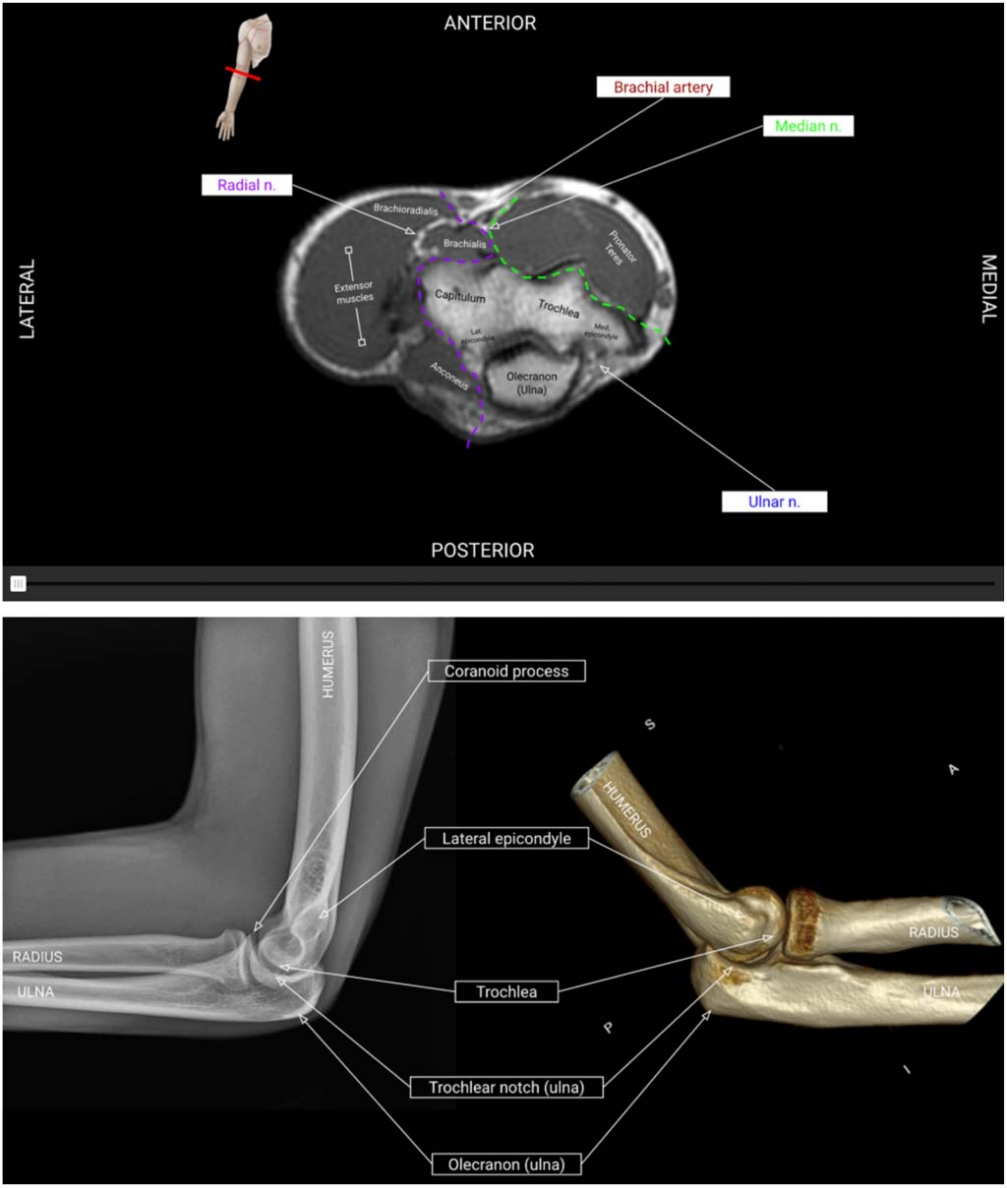

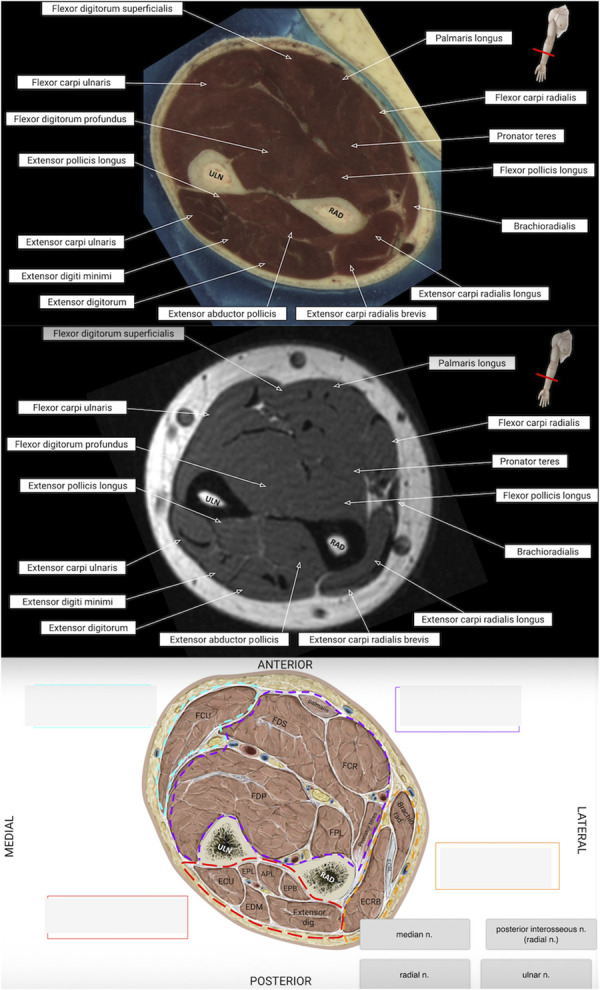

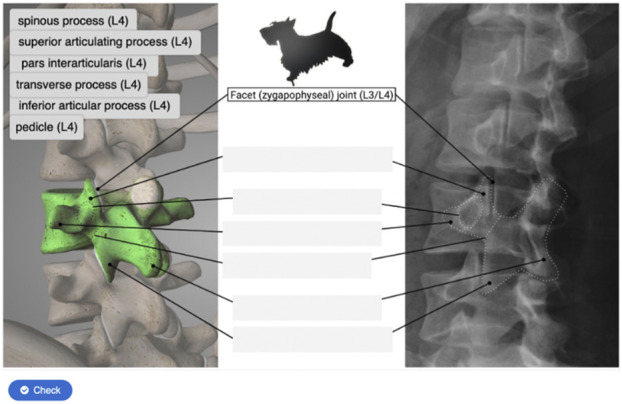
**Fig. 1-A** Comparison of bony landmarks on a CT model vs. x-ray model of the elbow. **Fig. 1-B** Interactive interfaces to visualize illustrational, MRI, and cadaveric cross-sections. **Fig. 1-C**. Correlating structures on 3D model illustrations and radiographs. CT = computed tomography, and MRI = magnetic resonance imaging.

**Fig. 2:**
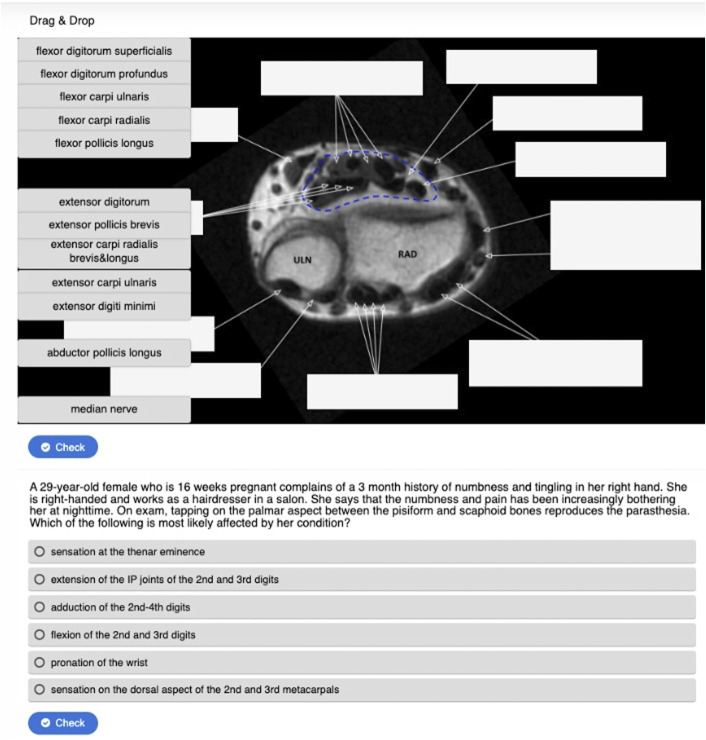
Example of an interactive recall exercise with MRI anatomy and a related multiple-choice question within the upper limb module. MRI, magnetic resonance imaging.

The upper extremity module is organized by shoulder, upper arm, elbow, forearms, wrist, and hand. The spine module is separated into cervical, thoracic, and lumbar spine. The modules allow users to interact with 3 types of cross-sectional imaging juxtaposed: cadaveric sections from the Human Visibility Project, axial computed tomography (CT) and magnetic resonance imaging (MRI) images, and cross-sectional anatomic illustrations^8^. They also incorporate images of cadaveric dissections and radiographs (Figs. 1-A, 1-B, and 1-C). Users are guided through muscular, skeletal, nervous, and vascular anatomy and spatial relationships using images and interactive interfaces to support multiperspective visualization. The module requires learners to identify and label structures, reinforcing pathoanatomy and clinical associations through active recall. There are vignette-style practice questions including imaging at the end of each module (Fig. 2). A postmodule assessment is provided at the conclusion of the module.

Each module is self-paced and would take between 30 and 60 minutes to complete. The modules were made available to students enrolled in the MSK course as an optional resource to use before the end-of-course assessment, with no associated incentives for participation.

The control group consisted of students who did not complete the supplemental MSK modules and instead only participated in the existing MSK course. This course includes 40 traditional lecture sessions (including 1 session on MSK radiology), 2 interactive discussion sessions, and 2 faculty-guided cadaver labs.

### Assessment and Data Collection

**Fig. 3:**
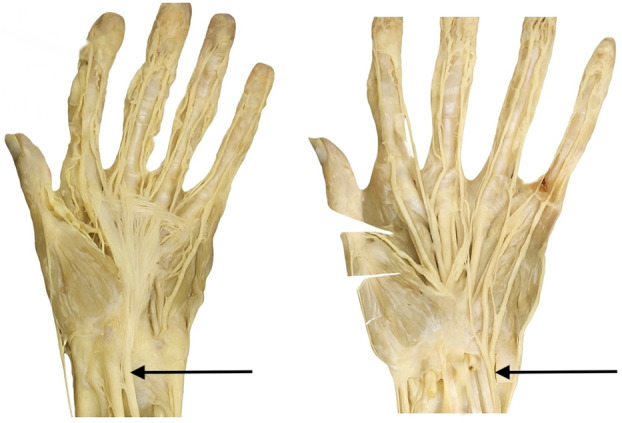
Two questions from the anatomy practical exam: (Left) Identify the tendon indicated by the black arrow. (Right) Identify the nerve indicated by the black arrow.

Two end-of-course assessments are in place at the conclusion of the MSK course, a written examination with multiple choice questions and practical anatomy assessment. The practical anatomy examination is organized by cadaver station that tags 2 structures for each cadaver, including upper limb, spine, and lower limb muscles, in addition to neurovascular structures (Fig. 3). Tags can vary year-to-year but are based on a consistent list of core anatomic structures. The written examination remains largely the same from year-to-year, with minor variations of a few questions.

Student engagement data from 2022 to 2025 included individual module scores and time spent completing modules. MSK anatomy practical and written examination performance was compared between module users and nonusers. In the 2023 academic year only, module completers were offered an optional postmodule survey assessing perceptions of the upper limb and spine modules. Each module survey had 7 questions comparing its perceived effectiveness to traditional teaching methods, including cadaver laboratory and lectures, focusing on clarity, usefulness, and overall learning benefit.

Statistical analyses included unequal variance two-sample *t* tests for continuous variables and χ^2^ tests for categorical variables. A post hoc power analysis was conducted for both all-years of the upper extremity and spine modules and a stratified analysis of year-by-year for written and practical examinations. A nominal alpha of 0.00625 was used for the power analysis, with a value of greater than 0.8 deemed as adequate power. A p < 0.05 was considered statistically significant.

## Results

### Participant Engagement and Assessment Performance

Between the academic years of 2022 and 2025, data were collected from 488 second-year medical students enrolled in the MSK course. A total of 114 students completed the spine module, and 165 students completed the upper limb module, representing a participation rate of 23.5% and 33.8%, respectively (Table I, Supplementary Table I).

**TABLE I:** Modules Completion Status and Examination Scores, Spine Module

Final practical grade	No	Yes	p
n	374	114	p < 0.0001
Mean(SD)	88.5 (9.50)	92 (8.46)	—
Median	89.75	96	—
Range	20.0–100.0	60.0–100.0	—
Final written grade	No	Yes	p
n	374	114	p = 1.0
Mean(SD)	85.1 (8.1)	85.1 (8.3)	—
Median	85.6	86.5	—
Range	43.5–100.0	56.5–96.0	—

Those who completed the spine anatomy modules from 2022 to 2025 had a mean 3.5% increase in the practical anatomy final examination scores (p < 0.0001), from those who did not complete the modules with a mean of 88.5% (±9.50), and those who did complete the modules with a mean of 92.0%(±8.46). Those who completed the spine anatomy modules had a mean 0% increase in written final examination scores. Students who completed the upper extremity module had an overall mean increase of 3.3% of their final practical examination score, which reached statistical significance (p = 0.00025). **Those who did not complete the upper extremity modules had a mean of 88.2%(±10.1) and those who did complete the modules had a mean of 91.5%(±8.9).** Those who did not complete the upper limb modules had a mean final written examination score of 84.9% (±8.2), while those who completed the module had a mean score of 85.7 (±7.8); however, this difference of 0.8% did not reach statistical significance (p = 0.293). A year-by-year analysis was completed, represented in Supplementary Table 2.

### Subjective Student Survey

Responses were collected and analyzed from the optional postmodule survey in the **2023 academic year** (Table II). There were 30 responses (79% response rate from module users) recorded for the spine and 26 responses (57% response rate) from the upper limb postmodule survey. 98.0% of respondents strongly agreed or agreed that the module was effective in improving their understanding of MSK anatomy of the upper limb. 49 (96.1%) respondents strongly agreed or agreed with the statement “Learning anatomy in this format will help me better assess pathology in patients in the future.” For the statement “Cadaver laboratory instruction is more effective than module-based learning for learning anatomy,” 16 (34.8%) respondents either strongly disagreed, disagreed, or were undecided. For the statement “Didactic instruction (lectures) is more effective than module-based learning for learning anatomy”; 19 (36.5%) respondents either strongly agreed or agreed. Therefore, students reported that module-based learning enhanced their understanding of anatomy compared with traditional lectures, but it did not surpass or replace the value of cadaveric instruction.

**TABLE II:** Post-Module Surveys

	Total: 50
This module was effective in improving your understanding of MSK anatomy of the upper limb, n (%)	
Disagree	1 (2.0%)
Agree	15 (29.4%)
Strongly agree	34 (66.6%)
	Total: 46
Cadaver lab instruction is more effective than module-based learning for learning anatomy, n (%)	
Strongly disagree	5 (10.9%)
Disagree	6 (13.0%)
Undecided	5 (10.9%)
Agree	12 (26.1%)
Strongly agree	18 (39.1%)
	Total: 52
Didactic instruction (lectures) is more effective than module-based learning for learning anatomy, n (%)	
Strongly disagree	4 (7.7%)
Disagree	22 (42.3%)
Undecided	7 (13.5%)
Agree	8 (15.4%)
Strongly agree	11 (21.1%)
	Total: 51
Learning anatomy in this format will help me better assess pathology in patients in the future, n (%)	
Undecided	2 (3.9%)
Agree	24 (47.1%)
Strongly agree	25 (49.0%)
	Total: 51
This module increased your preparedness for your final course exams, n (%)	
Undecided	1 (4.8%)
Agree	4 (19.0%)
Strongly agree	16 (76.2%)

MSK = musculoskeletal.

Power analysis for all-year written and practical examination scores for students who took the upper extremity or spine modules had values of 0.827 and 0.964, respectively, demonstrating that the study was adequately powered over 4 years. The power for individual years of 2022, 2023, 2024, and 2025 for written and practical examination scores for upper extremity and spine modules were all below 0.4.

## Discussion

This study found that the usage of 2 online learning modules that integrated MSK anatomy content with comparative radiologic and cross-sectional imaging and analyzed improved end-of-course MSK practical examination scores. Interestingly, while practical scores improved, written examination performance remained similar between module users and nonusers. This suggests that radiological integration may specifically enhance three-dimensional spatial reasoning, which practical examinations capture more effectively. The written examination evaluates conceptual, recall-based MSK knowledge, including pathophysiology, pharmacology, microbiology, and embryology. This contrasts with the visuospatial demands of the anatomy practical, making the practical examination an appropriate measure of MSK anatomy comprehension while the written examination serves as a comparison dataset that helps control for motivational factors.

Teaching anatomy through radiology is perhaps effective because collapsing complex 3-dimensional structures into 2-dimensional snapshots directs learners conceptually reconcile anatomical arrangement and function^9^. Compared with gross dissection, CT and MRI reveal a more appreciable representation of the muscles, layering, neurovasculature, structural relationships, and spatial distribution. Providing a visual comparison with the Human Visibility Project images allows learners to appreciate the rendering of the cadaveric anatomy in CT and MRI imaging. Chew et al. reported that adding cadaveric CT scans to the standard anatomy curriculum significantly improved student test scores, demonstrating the value of imaging integration for enhancing spatial comprehension^10^.

Previous studies have consistently supported integrating radiology into anatomy education. Chew et al. demonstrated that adding small-group radiology sessions to a first-year curriculum significantly improved examination scores^11^. Other investigations, including those by Philips & Kumar, found that self-directed radiology modules enhanced anatomy examination performance and were strongly recommended by students as effective learning tools^12,13^. Similarly, Rathan et al. and Rajprasath et al. reported that incorporating radiological images into assessments or teaching sessions improved anatomical understanding and facilitated application in clinical contexts^14,15^.

Self-directed, module-based learning promotes active engagement and allows students to reason through spatial relationships at their own pace. Studies by Nagaraj and Grignon both demonstrated that radiology-integrated modules improved anatomy knowledge and examination performance^16,17^. These findings highlight the value of asynchronous, imaging-enhanced learning as a complement to cadaveric dissection and traditional teaching.

Our survey results suggest that most students found the modules significantly enhanced upper limb and spine anatomy knowledge compared with lectures, although they did not replace cadaveric instruction. This supports using radiology and cross-sectional imaging as an adjunct to traditional dissection, consistent with prior literature. Murphy et al. surveyed first-year medical students and found that cadaveric dissection was ranked as the most valuable instructional format, followed by supplemental interactive sessions incorporating radiology with anatomy, and, lastly, lecture^18^. Similarly, Phillips et al. conducted a randomized trial comparing radiology-cadaver correlation sessions to standard dissection^19^. Students rated the radiology sessions as highly as cadaveric dissection, supporting their use as complementary tools in a blended curriculum rather than stand-alone methods.

A potential limitation of this study is that students who completed the modules may have been inherently more motivated, diligent, or interested, which could introduce selection bias. Nevertheless, improvements were observed specifically on the practical examination, which evaluates spatial and visual understanding, rather than on the written examination. In addition, the inclusion of a large cohort spanning 4 academic years enhances the robustness of the findings and helps mitigate the impact of this potential bias. Ultimately, our call-to-action is not to suggest that every curriculum immediately implement this type of cross-sectional radiology integration. Students have diverse learning styles and providing additional resources in MSK education is beneficial. Our combined objective and subjective data support the inclusion of this learning modality. Future studies could further explore this modality by using multicenter randomized assignment, stratifying learners based on baseline performance, assessing clinical application, and assessing long-term retention.

Integration of radiology-based anatomy resources into medical education may have broader implications beyond the preclinical setting. This approach could also be applied to residency training, particularly in orthopaedic surgery and radiology, where correlating imaging with anatomy is critical for diagnosis and preoperative planning. Comparing resident performance on radiology evaluations with anatomical knowledge and surgical skills may further clarify how imaging competency relates to operative ability.

## Conclusion

Integrating radiologic and cross-sectional imaging into preclinical MSK anatomy education significantly improved practical anatomy performance without altering written examination outcomes. Subjective survey data demonstrated high student satisfaction and perceived efficacy of the modality. Together, these findings support imaging-based, self-directed modules as valuable adjuncts to cadaveric dissection and didactic sessions, as they can strengthen visuospatial reasoning and better prepare trainees for clinical application.

## Appendix

Supporting material provided by the authors is posted with the online version of this article as a data supplement at jbjs.org (http://links.lww.com/JBJSOA/B201). This content was not copyedited or verified by JBJS.
